# Prevalence of germline *BRCA* mutations in HER2-negative metastatic breast cancer: global results from the real-world, observational BREAKOUT study

**DOI:** 10.1186/s13058-020-01349-9

**Published:** 2020-10-27

**Authors:** Joyce O’Shaughnessy, Christine Brezden-Masley, Marina Cazzaniga, Tapashi Dalvi, Graham Walker, James Bennett, Shozo Ohsumi

**Affiliations:** 1 Baylor University Medical Center, Texas Oncology and US Oncology, Dallas, TX USA; 2 Cancer Program at Sinai Health System, Toronto, Ontario Canada; 3 Ospedale San Gerardo Monza, Monza, Italy; 4 AstraZeneca Pharmaceuticals, LP, Gaithersburg, MD USA; 5 AstraZeneca, Cambridge, UK; 6 NHO Shikoku Cancer Center, Matsuyama-shi, Ehime-Ken Japan

**Keywords:** Breast cancer susceptibility genes, *BRCA*, Prevalence, Observational

## Abstract

**Background:**

The global observational BREAKOUT study investigated germline *BRCA* mutation (gBRCAm) prevalence in a population of patients with human epidermal growth factor receptor 2 (HER2)-negative metastatic breast cancer (MBC).

**Methods:**

Eligible patients had initiated first-line cytotoxic chemotherapy for HER2-negative MBC within 90 days prior to enrollment. Hormone receptor (HR)-positive patients had experienced disease progression on or after prior endocrine therapy, or endocrine therapy was considered unsuitable. gBRCAm status was determined using baseline blood samples or prior germline test results. For patients with a negative gBRCAm test, archival tissue was tested for somatic BRCAm and homologous recombination repair mutations (HRRm). Details of first-line cytotoxic chemotherapy were also collected.

**Results:**

Between March 2017 and April 2018, 384 patients from 14 countries were screened and consented to study enrollment; 341 patients were included in the full analysis set (median [range] age at enrollment: 56 [25–89] years; 256 (75.3%) postmenopausal). Overall, 33 patients (9.7%) had a gBRCAm (16 [4.7%] in g*BRCA1* only, 12 [3.5%] in g*BRCA2* only, and 5 [1.5%] in both g*BRCA1* and g*BRCA2*). gBRCAm prevalence was similar in HR-positive and HR-negative patients. gBRCAm prevalence was 9.0% in European patients and 10.6% in Asian patients and was higher in patients aged ≤ 50 years at initial breast cancer (BC) diagnosis (12.9%) than patients aged > 50 years (5.4%). In patients with any risk factor for having a gBRCAm (family history of BC and/or ovarian cancer, aged ≤ 50 years at initial BC diagnosis, or triple-negative BC), prevalence was 10.4%, versus 5.8% in patients without these risk factors. HRRm prevalence was 14.1% (*n* = 9/64) in patients with germline *BRCA* wildtype.

**Conclusions:**

Patient demographic and disease characteristics supported the association of a gBRCAm with younger age at initial BC diagnosis and family history of BC and/or ovarian cancer. gBRCAm prevalence in this cohort, not selected on the basis of risk factors for gBRCAm, was slightly higher than previous results suggested. gBRCAm prevalence among patients without a traditional risk factor for harboring a gBRCAm (5.8%) supports current guideline recommendations of routine gBRCAm testing in HER2-negative MBC, as these patients may benefit from poly(ADP-ribose) polymerase (PARP) inhibitor therapy.

**Trial registration:**

NCT03078036.

## Background

Breast cancer is the most commonly diagnosed cancer in women (excluding non-melanoma skin cancers) and is the most common cause of cancer deaths in women worldwide [1]. The breast cancer susceptibility genes (*BRCA1* and *BRCA2*) encode proteins critically involved in the repair of DNA double-strand breaks [2]. A germline *BRCA1* and/or *BRCA2* mutation (gBRCAm) substantially increases the risk of developing breast and/or ovarian cancer, as well as other tumor types such as prostate and pancreatic cancer [3–7].

Poly(ADP-ribose) polymerase (PARP) inhibitors block DNA damage repair in cells harboring a deficiency in homologous recombination repair (HRR), including mutations in *BRCA1* and *BRCA2* [8]. The PARP inhibitors olaparib and talazoparib have proven effective at targeting *BRCA*-mutated human epidermal growth factor receptor 2 (HER2)-negative metastatic breast cancers, including in the Phase 3 OlympiAD (NCT02000622) and EMBRACA (NCT01945775) trials [9–12]. The prevalence of a gBRCAm ranges from 1.2 to 8.8% in unselected breast cancer patient populations [13–19]. However, limited data exist on the prevalence of a gBRCAm in patients with HER2-negative metastatic breast cancer who require treatment with a first-line systemic chemotherapy regimen. Such data would provide an estimate of the size of the patient population who may be appropriate candidates to receive treatment with a PARP inhibitor for gBRCAm metastatic breast cancer.

Here, we present data from the global observational BREAKOUT study (NCT03078036) in patients with HER2-negative metastatic breast cancer being treated with first-line chemotherapy, the primary objective of which was to estimate the prevalence of gBRCAm in this patient population.

## Methods

### Study design

**Fig. 1:**
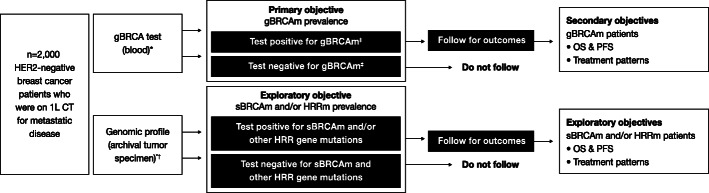
BREAKOUT study design. *BRCA*, breast cancer susceptibility gene; CT, chemotherapy; gBRCAm, germline *BRCA* mutation; HER2, human epidermal growth factor receptor-2; HRRm, homologous recombination repair gene mutation; OS, overall survival; PFS, progression-free survival; sBRCAm, somatic *BRCA* mutation. *Blood/tumor testing occurred concurrently to the extent possible. ^†^Foundation Medicine Inc. (Cambridge, Massachusetts, USA) Lynparza HRR assay was used to test for HRR gene mutations. ^‡^Positive: deleterious mutation; suspected deleterious. Negative: no deleterious mutation detected; no mutation detected; favor polymorphism; variant of uncertain significance; *BRCA* wildtype

BREAKOUT was an observational, cross-sectional study, with a nested prospective cohort component (Fig. 1). The study design was based on the Phase 3b real-world, open-label, single-arm LUCY study (NCT03286842) [20]. In the LUCY study, patients with a gBRCAm HER2-negative metastatic breast cancer were being treated with olaparib following no more than two prior lines of chemotherapy in the metastatic setting. BREAKOUT study sites were selected for their willingness to participate in the study and were requested to enroll sequential patients with HER2-negative metastatic breast cancer. See Additional file 1 for a list of participating study sites. The primary objective of BREAKOUT was to assess the prevalence of gBRCAm in a global population of patients with HER2-negative metastatic breast cancer who were not selected on the basis of risk factors for gBRCAm. For patients who tested positive for a gBRCAm, the planned secondary objectives included assessment of treatment patterns by line of therapy and prospective evaluation of clinical outcomes, including progression-free survival and overall survival.

In order to minimize selection bias, patients were selected regardless of demographic characteristics, presence of risk factors for gBRCAm breast cancer, or previously known gBRCAm status.

For patients who tested negative for a gBRCAm (gBRCAwt), archival tumor specimens were obtained where possible, to test for somatic *BRCA* mutations (sBRCAm) and other HRR gene mutations, using an investigational clinical trial assay, based on the FoundationOne® CDx platform (Foundation Medicine Inc., Cambridge, Massachusetts, USA). The HRR genes evaluated were *BRCA1*, *BRCA2*, *ATM*, *RAD51B*, *RAD51C*, *RAD51D*, *RAD54L*, *BRIP1*, *FANCL*, *PALB2*, *BARD1*, *CHEK1*, *CHEK2*, *CDK12*, and *PPP2R2A*.

The planned sample size was 2000 patients with HER2-negative metastatic breast cancer. Study enrollment was terminated early due to poor recruitment and, therefore, only patients’ first-line cytotoxic chemotherapy regimens were collected for analysis. Data on subsequent therapies for metastatic breast cancer, progression-free survival, and overall survival were not collected due to the limited number of patients and absence of follow-up data.

Data including baseline patient demographic and disease characteristics, medical history, and treatment history were collected from individual patient records. Family history of breast and/or ovarian cancer in first- or second-degree relatives was also collected. Patients’ gBRCAm status was determined by testing baseline blood samples (in a central laboratory, or in Japan using the Myriad BRACAnalysis CDx® test) or, where available, using existing gBRCAm test results that had been obtained by local or central testing, as per local practice.

gBRCAm results were classified as positive (deleterious g*BRCA1* and/or g*BRCA2* mutation; genetic variant suspected deleterious), negative (gBRCAwt; genetic variant of uncertain significance; genetic variant, favor polymorphism; no mutation/deleterious mutation detected), or not determined.

### Patient population

Eligible patients had started a first-line chemotherapy regimen for HER2-negative metastatic breast cancer within 90 days prior to enrollment. First-line chemotherapy was defined as the first chemotherapy given in the metastatic setting. Patients with hormone receptor (HR)-positive metastatic breast cancer had developed disease progression on or after prior endocrine therapy, or were considered unsuitable for endocrine therapy. Patients who had received prior PARP inhibitor therapy were not eligible for the study.

### Statistical analysis

The full analysis set (FAS) comprised all enrolled, eligible patients who had available gBRCAm test results. An exploratory subgroup included all patients in the FAS who had been tested for sBRCAm and/or HRR gene mutations, including those whose status could not be determined (e.g., due to the sample not being evaluable).

The prevalence of a gBRCAm was determined using the number of patients who were tested and had a valid result for gBRCAm status as the denominator and the number of patients who were gBRCAm-positive as the numerator. The prevalence of a gBRCAm according to demographic variables, disease characteristics, comorbidities, and choice of first-line therapy regimens was also evaluated.

Appropriate descriptive statistics were provided for continuous and categorical variables.

## Results

**Fig. 2:**
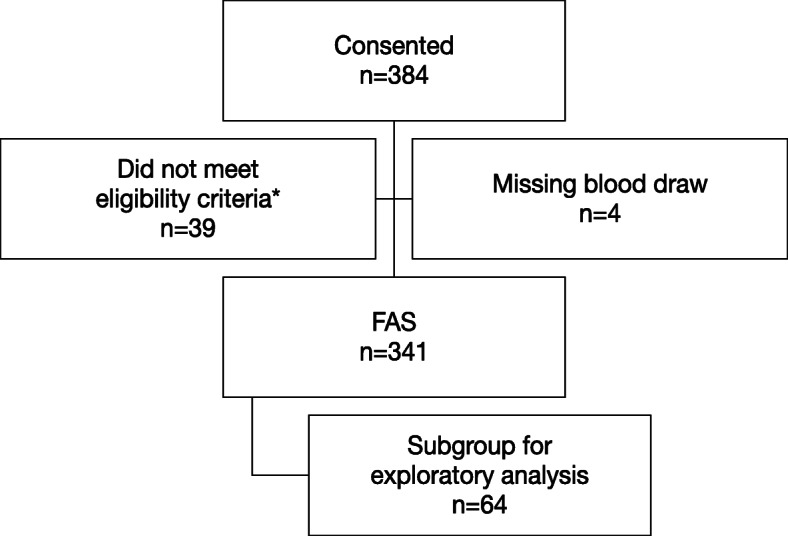
Patient disposition. FAS, full analysis set. *39 patients did not meet eligibility criteria; 29 patients had not initiated treatment with first-line systemic cytotoxic chemotherapy for metastatic breast cancer in the past 90 days and, at that time, were considered to have exhausted endocrine therapy options if hormone receptor-positive; 9 patients had no evidence of metastatic disease; and 1 patient consented after the termination of the study

Between March 2017 and April 2018, 384 patients were screened and consented to study enrollment; 43 patients were excluded from the analysis—4 for whom a blood sample for gBRCAm testing was not available and 39 for not meeting prespecified eligibility criteria—resulting in 341 patients with HER2-negative metastatic breast cancer comprising the FAS (Fig. 2).

Patients were enrolled across 14 countries: Australia (*n* = 5), Bulgaria (*n* = 15), Canada (*n* = 8), Hungary (*n* = 3), Italy (*n* = 7), Japan (*n* = 44), Poland (*n* = 13), Russia (*n* = 32), South Korea (*n* = 45), Spain (*n* = 18), Taiwan (*n* = 15), Turkey (*n* = 79), the UK (*n* = 32), and the USA (*n* = 25).

### gBRCAm prevalence

Of the 341 patients included in the FAS, 33 (9.7%; 95% confidence interval [CI] 6.8%, 13.3%) harbored a gBRCAm (Table 1). Mutations were detected in g*BRCA1* alone in 16 patients (4.7%; 95% CI 2.7%, 7.5%), in g*BRCA2* alone in 12 patients (3.5%; 95% CI 1.8%, 6.1%), and in both g*BRCA1* and g*BRCA2* in 5 patients (1.5%; 95% CI 0.5%, 3.4%). A total of 30 patients underwent gBRCAm testing prior to baseline; 8 (26.7%) of these patients had a gBRCAm.

**Table 1:** gBRCAm prevalence by region of enrollment (FAS)

	Asia (***N*** = 104)	Europe (***N*** = 199)	North America (***N*** = 33)	Australia/Oceania (***N*** = 5)	FAS (***N*** = 341)
Positive for a gBRCAm, *n* (% [95% CI])	11 (10.6 [5.4, 18.1])	18 (9.0 [5.4, 13.9])	3 (9.1 [1.9, 24.3])	1 (20.0 [0.5, 71.6])	33 (9.7 [6.8, 13.3])
g*BRCA1*m only, *n* (% [95% CI])	6 (5.8 [2.1, 12.1])	10 (5.0 [2.4, 9.0])	0 (0.0 [0.0, 10.6])	0 (0.0 [0.0, 52.2])	16 (4.7 [2.7, 7.5])
g*BRCA2*m only, *n* (% [95% CI])	5 (4.8 [1.6, 10.9])	6 (3.0 [1.1, 6.4])	1 (3.0 [0.1, 15.8])	0 (0.0 [0.0, 52.2])	12 (3.5 [1.8, 6.1])
Both g*BRCA1*m and g*BRCA2*m, *n* (% [95% CI])	0 (0.0 [0.0, 3.5])	2 (1.0 [0.1, 3.6])	2 (6.1 [0.7, 20.2])	1 (20.0 [0.5, 71.6])	5 (1.5 [0.5, 3.4])

*BRCA* breast cancer susceptibility gene, *CI* confidence interval, *FAS* full analysis set, *gBRCAm* germline *BRCA* mutation

Subgroup analyses showed the prevalence of gBRCAm was 9.0% in European patients (*n* = 18/199; 95% CI 5.4%, 13.9%), 9.1% in North American patients (*n* = 3/33; 95% CI 1.9%, 24.3%), and 10.6% in Asian patients (Japan, South Korea, and Taiwan; *n* = 11/104; 95% CI 5.4%, 18.1%) (Table 1).

### s*BRCA* and HRR gene mutations

**Fig. 3:**
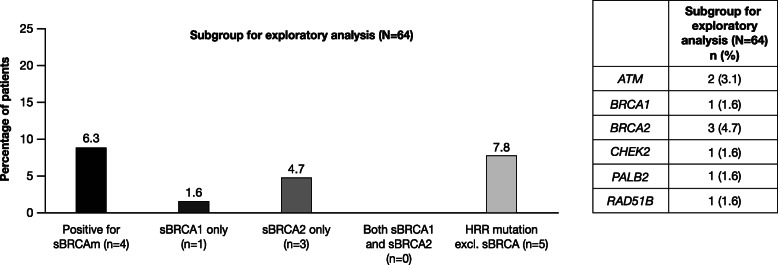
Prevalence of sBRCAm and other HRR gene mutations (subgroup for exploratory analysis). *BRCA1*, breast cancer susceptibility gene 1; *BRCA2*, breast cancer susceptibility gene 2; HRRm, homologous recombination repair gene mutation; sBRCAm, somatic *BRCA* mutation; s*BRCA1*, somatic *BRCA1* mutation; s*BRCA2*, somatic *BRCA2* mutation

In total, 64 patients with gBRCAwt who had archival breast cancer tissue were tested for HRR gene mutations and sBRCAm. The prevalence of sBRCAm was 6.3% (*n* = 4/64; 95% CI 1.7%, 15.2%). One patient had s*BRCA1* mutation only (1.6%; 95% CI 0.0%, 8.4%) and three patients had s*BRCA2* mutation only (4.7%; 95% CI 1.0%, 13.1%) (Fig. 3). The incidence of sBRCAm in patients with an existing gBRCAm was not assessed in the BREAKOUT study. The prevalence of any HRR gene mutation overall was 14.1% (*n* = 9/64; 95% CI 6.6%, 25.0%), including mutations in *ATM* (*n* = 2), *BRCA1* (*n* = 1), *BRCA2* (*n* = 3), *CHEK2* (*n* = 1), *PALB2* (*n* = 1), and *RAD51B* (*n* = 1) (Fig. 3).

### Patient demographic and clinical characteristics

Baseline demographic and clinical characteristics for patients in the FAS are shown in Table 2. Median (range) age at enrollment was 56 (25–89) years. Median (range) age at initial breast cancer diagnosis was 40 (24–71) years in patients with a gBRCAm and 52 (24–86) years in patients with gBRCAwt.

**Table 2:** Baseline patient demographic and disease characteristics (FAS)

	gBRCAm status		
	Positive (***N*** = 33)	Negative (***N*** = 308)	FAS (***N*** = 341)
Age at enrollment (years)			
*n*	33	308	341
Median (range)	47.0 (25–71)	56.5 (29–89)	56.0 (25–89)
Race, *n* (%)			
*n*	28	267	295
White	22 (78.6)	201 (75.3)	223 (75.6)
Black or African American	0	4 (1.5)	4 (1.4)
Asian	6 (21.4)	60 (22.5)	66 (22.4)
Native Hawaiian or Other Pacific Islander	0	1 (0.4)	1 (0.3)
Other	0	1 (0.4)	1 (0.3)
Age at initial breast cancer diagnosis (years)			
*n*	31	307	338
Median (range)	40.0 (24–71)	52.0 (24–86)	50.0 (24–86)
Family history of breast and/or ovarian cancer, *n* (%)			
*n*	33	307	340
Yes	15 (45.5)	51 (16.6)	66 (19.4)
No	18 (54.5)	256 (83.4)	274 (80.6)
Time since initial breast cancer diagnosis to enrollment (months)			
*n*	31	307	338
Median (interquartile range)	28.1 (13.4–73.0)	29.9 (6.3–78.2)	29.8 (7.2–76.8)
Sites of metastatic disease, *n* (%)			
*n*	33	308	341
Bone and locomotor	13 (39.4)	162 (52.6)	175 (51.3)
Lymph nodes	15 (45.5)	134 (43.5)	149 (43.7)
Respiratory	10 (30.3)	86 (27.9)	96 (28.2)
Liver	8 (24.2)	69 (22.4)	77 (22.6)
Other metastatic sites	23 (69.7)	194 (63.0)	217 (63.6)
HR status at most recent assessment, *n* (%)			
*n*	31	303	334
Positive	20 (64.5)	195 (64.4)	215 (64.4)
AJCC stage at initial breast cancer diagnosis, *n* (%)			
*n*	33	303	336
0	1 (3.0)	8 (2.6)	9 (2.7)
Stage I (I, A, B, C)	3 (9.1)	28 (9.2)	31 (9.2)
Stage II (II, A, B, C)	13 (39.4)	104 (34.3)	117 (34.8)
Stage III (III, A, B, C)	8 (24.2)	80 (26.4)	88 (26.2)
Stage IV (IV, A, B, C)	8 (24.2)	83 (27.4)	91 (27.1)
Nodal status at original diagnosis, *n* (%)			
*n*	33	307	340
N0	12 (36.4)	74 (24.1)	86 (25.3)
N1	8 (24.2)	106 (34.5)	114 (33.5)
N2	7 (21.2)	54 (17.5)	61 (17.9)
N3	5 (15.2)	38 (12.4)	43 (12.6)
pN0	0	4 (1.3)	4 (1.2)
NX	1 (3.0)	29 (9.4)	30 (8.8)
N1a	0	2 (0.7)	2 (0.6)
Tumor grade at original diagnosis, *n* (%)			
*n*	33	303	336
X (undetermined)	3 (9.1)	65 (21.5)	68 (20.2)
1 (well differentiated)	2 (6.1)	22 (7.3)	24 (7.1)
2 (moderately differentiated)	10 (30.3)	101 (33.3)	111 (33.0)
3 (poorly differentiated)	15 (45.5)	103 (34.0)	118 (35.1)
4 (undifferentiated)	0	3 (1.0)	3 (0.9)
High grade*	3 (9.1)	9 (3.0)	12 (3.6)
Non-chemotherapy treatment prior to metastatic disease, *n* (%)^†^			
*n*	33	305	338
Tamoxifen	7 (21.2)	74 (24.3)	81 (24.0)
Letrozole	3 (9.1)	37 (12.1)	40 (11.8)
Anastrozole	2 (6.1)	35 (11.5)	37 (10.9)
Exemestane	1 (3.0)	3 (1.0)	4 (1.2)
Fulvestrant	0	8 (2.6)	8 (2.4)
Everolimus	1 (3.0)	0	1 (0.3)
Other^‡^	1 (3.0)	11 (3.6)	12 (3.6)

*AJCC* American Joint Committee on Cancer, *BRCA* breast cancer susceptibility gene, *eCRF* electronic case report form, *FAS* full analysis set, *gBRCA* germline *BRCA* mutation, *HR* hormone receptor, *SD* standard deviation

*High grade was listed as an additional category in the eCRF and is based on the Nottingham grading system (total score: 8–9)

^†^A patient may have had more than one type of non-chemotherapy treatment

^‡^Additional non-chemotherapy treatments to those listed in the table included leuprorelin/leuprorelin acetate (*n* = 5), toremifene/toremifene citrate (*n* = 3), bevacizumab (*n* = 1), goserelin (*n* = 1), tamoxifen citrate (*n* = 1), and trastuzumab (*n* = 1)

Most patients were postmenopausal at enrollment (*n* = 256/340; 75.3%). The proportion of postmenopausal patients was lower in the gBRCAm group (57.6%) compared with the gBRCAwt group (77.2%). In the FAS, 58.1% of patients (*n* = 198/341) were postmenopausal at initial breast cancer diagnosis. Most patients with gBRCAwt were postmenopausal at initial breast cancer diagnosis (*n* = 186/308; 60.4%), compared with 36.4% of patients with a gBRCAm (*n* = 12/33).

Median time from initial breast cancer diagnosis to enrollment was 28.1 months (interquartile range 13.4–73.0) in patients with a gBRCAm and was similar at 29.9 months (interquartile range 6.3–78.2) in patients with gBRCAwt (Table 2).

Disease stage at initial breast cancer diagnosis in the FAS was stage 0 in 2.7% (*n* = 9/336) of patients, stage I in 9.2% (31/336) of patients, stage II in 34.8% (*n* = 117/336) of patients, stage III in 26.2% (*n* = 88/336) of patients, and stage IV in 27.1% (91/336) of patients, with similar distribution among patients with a gBRCAm (Table 2). With respect to nodal status at initial breast cancer diagnosis, in the FAS, 25.3% of patients had node stage N0 (negative) and 33.5% had node stage N1 disease. Tumor grade at initial breast cancer diagnosis was poorly differentiated in 35.1% of patients (*n* = 118/336) and moderately differentiated in 33.0% of patients (*n* = 111/336) (Table 2). In comparison with all patients in the FAS, more patients with a gBRCAm had poorly differentiated cancers (45.5%; *n* = 15/33) and 30.3% (*n* = 10/33) of patients with a gBRCAm had moderately differentiated tumors.

Overall, 52.0% (*n* = 156/300) of patients with gBRCAwt had received chemotherapy prior to metastatic disease, compared with 64.5% (*n* = 20/31) of patients with a gBRCAm, and 39.0% (*n* = 119/305) of patients with gBRCAwt received non-chemotherapy treatments prior to metastatic disease, compared with 30.3% (*n* = 10/33) of patients with a gBRCAm. Non-chemotherapy treatments included endocrine therapy (including tamoxifen, letrozole, anastrozole, exemestane, and fulvestrant), everolimus (*n* = 1), and bevacizumab (*n* = 1) (Table 2) and were mostly (77.9%) used as adjuvant therapy.

Non-chemotherapy treatments (including letrozole, fulvestrant, and exemestane) were administered for metastatic disease prior to receiving first-line chemotherapy in 24.2% (*n* = 8/33) of patients with a gBRCAm and 30.9% (*n* = 95/307) of patients with gBRCAwt.

Similar proportions of patients with a gBRCAm or gBRCAwt had HR-positive (estrogen receptor- and/or progesterone receptor-positive) versus HR-negative HER2-negative metastatic breast cancer with gBRCAwt (Table 2).

A family history of breast and/or ovarian cancer in a first- or second-degree relative was recorded for 45.5% (*n* = 15/33) of patients with a gBRCAm, compared with 16.6% (*n* = 51/307) of patients with gBRCAwt (Table 2).

### Subgroup analyses by risk factors for gBRCAm

#### Age at initial breast cancer diagnosis

The prevalence of a gBRCAm was 12.9% (*n* = 22/171; 95% CI 8.2%, 18.8%) in patients aged ≤ 50 years at initial breast cancer diagnosis and was lower at 5.4% (*n* = 9/167; 95% CI 2.5%, 10.0%) in patients > 50 years of age at initial breast cancer diagnosis (Table 3). The prevalence of a g*BRCA1* mutation was higher in patients aged ≤ 50 years at initial breast cancer diagnosis (7.6%; *n* = 13/171; 95% CI 4.1%, 12.6%) than in patients aged > 50 years at initial breast cancer diagnosis (1.2%; *n* = 2/167; 95% CI 0.1%, 4.3%), whereas prevalence of a g*BRCA2* mutation was similar in patients aged ≤ 50 years and > 50 years, at 3.5% (95% CI 1.3%, 7.5%) and 3.0% (95% CI 1.0%, 6.8%), respectively.

**Table 3:** gBRCAm prevalence by risk factors for gBRCAm (FAS)

Risk factor	Positive for a gBRCAm, ***n*** (% [95% CI])	g***BRCA1***m only, ***n*** (% [95% CI])	g***BRCA2***m only, ***n*** (% [95% CI])	Both g***BRCA1***m and g***BRCA2***m, ***n*** (% [95% CI])
**Age at initial breast cancer diagnosis**				
≤ 50 years (*N* = 171)	22 (12.9 [8.2, 18.8])	13 (7.6 [4.1, 12.6])	6 (3.5 [1.3, 7.5])	3 (1.8 [0.4, 5.0])
> 50 years (*N* = 167)	9 (5.4 [2.5, 10.0])	2 (1.2 [0.1, 4.3])	5 (3.0 [1.0, 6.8])	2 (1.2 [0.1, 4.3])
**HR status**				
HR-positive (*N* = 215)	20 (9.3 [5.8, 14.0])	6 (2.8 [1.0, 6.0])	10 (4.7 [2.3, 8.4])	4 (1.9 [0.5, 4.7])
HR-negative (N = 119)	11 (9.2 [4.7, 15.9])	9 (7.6 [3.5, 13.9])	2 (1.7 [0.2, 5.9])	0 (0.0 [0.0, 3.1])
**Family history of breast and/or ovarian cancer**				
Yes (*N* = 66)	15 (22.7 [13.3, 34.7])	8 (12.1 [5.4, 22.5])	6 (9.1 [3.4, 18.7])	1 (1.5 [0.0, 8.2])
No (*N*= 274)	18 (6.6 [3.9, 10.2])	8 (2.9 [1.3, 5.7])	6 (2.2 [0.8, 4.7])	4 (1.5 [0.4, 3.7])

*BRCA* breast cancer susceptibility gene, *CI* confidence interval, *FAS* full analysis set, *gBRCAm* germline *BRCA* mutation, *HR* hormone receptor

#### Hormone receptor status

The overall prevalence of a gBRCAm was similar with respect to HR status: 9.3% (*n* = 20/215; 95% CI 5.8%, 14.0%) in patients with HR-positive disease (6 patients in g*BRCA1* only, 10 in g*BRCA2* only, and 4 in both g*BRCA1* and g*BRCA2*) and 9.2% (*n* = 11/119; 95% CI 4.7%, 15.9%) in patients with HR-negative disease (9 patients in g*BRCA1* only and 2 in g*BRCA2* only) (Table 3). However, the prevalence of a g*BRCA1* mutation was 7.6% (95% CI 3.5%, 13.9%) in patients with HR-negative metastatic breast cancer and 2.8% (95% CI 1.0%, 6.0%) in patients with HR-positive metastatic breast cancer, while a g*BRCA2* mutation was more frequent in those with HR-positive metastatic breast cancer, compared with HR-negative metastatic breast cancer (4.7% [95% CI 2.3%, 8.4%] and 1.7% [95% CI 0.2%, 5.9%], respectively).

#### Family history of breast and/or ovarian cancer

The prevalence of a gBRCAm was higher (22.7%; *n* = 15/66; 95% CI 13.3%, 34.7%) in the subgroup of patients with a family history of breast and/or ovarian cancer, compared with patients without a family history of breast and/or ovarian cancer (6.6%; *n* = 18/274; 95% CI 3.9%, 10.2%) (Table 3). In patients with a family history of breast and/or ovarian cancer, mutations in g*BRCA1* (12.1% [95% CI 5.4%, 22.5%]) and g*BRCA2* (9.1% [95% CI 3.4%, 18.7%]) were more prevalent than in patients without a family history of breast and/or ovarian cancer (2.9% [95% CI 1.3%, 5.7%] and 2.2% [95% CI 0.8%, 4.7%], respectively).

#### Presence of ≥ 1 risk factor for having a gBRCAm

gBRCAm prevalence was 10.4% (*n* = 26/250; 95% CI 6.9%, 14.9%) in patients who had at least one risk factor for having a gBRCAm (family history of breast and/or ovarian cancer; age at initial breast cancer diagnosis ≤ 50 years; or triple-negative breast cancer), compared with 5.8% (*n* = 5/86; 95% CI 1.9%, 13.0%) in patients without any of these risk factors.

### First-line chemotherapy use

Overall, most patients in the FAS received single-agent chemotherapy as their first-line chemotherapy regimen (*n* = 196/341; 57.5%) (Table 4), while 54.5% (*n* = 18/33) of patients with a gBRCAm received combination chemotherapy with ≥ 2 agents as their first-line chemotherapy regimen. Overall, the most frequently used single-agent regimens (> 10%) were paclitaxel (*n* = 75/196; 38.3%), capecitabine (*n* = 42/196; 21.4%), and docetaxel (*n* = 24/196; 12.2%), while the most frequently used combination regimen was paclitaxel/bevacizumab (*n* = 21/145; 14.5%) (see Additional file 2).

**Table 4:** Cytotoxic chemotherapies administered as first-line therapy for metastatic breast cancer (FAS)

	gBRCAm status		
	Positive (***N*** = 33)	Negative (***N*** = 308)	FAS (***N*** = 341)
Number of unique agents received as first-line therapy, *n* (%)*			
1	15 (45.5)	181 (58.8)	196 (57.5)
2	15 (45.5)	105 (34.1)	120 (35.2)
3	1 (3.0)	16 (5.2)	17 (5.0)
4+	2 (6.1)	6 (1.9)	8 (2.3)
Cytotoxic chemotherapy agent (in > 5% of patients), *n* (%)*			
Paclitaxel	12 (36.4)	115 (37.3)	127 (37.2)
Cyclophosphamide	5 (15.2)	55 (17.9)	60 (17.6)
Capecitabine	7 (21.2)	50 (16.2)	57 (16.7)
Docetaxel	6 (18.2)	42 (13.6)	48 (14.1)
Carboplatin	5 (15.2)	26 (8.4)	31 (9.1)
Doxorubicin	3 (9.1)	26 (8.4)	29 (8.5)
Gemcitabine	4 (12.1)	24 (7.8)	28 (8.2)
Bevacizumab	4 (12.1)	22 (7.1)	26 (7.6)
Cisplatin	1 (3.0)	22 (7.1)	23 (6.7)
Epirubicin	2 (6.1)	19 (6.2)	21 (6.2)

First-line cytotoxic chemotherapy was defined as the first chemotherapy given in the metastatic setting up to disease progression. The first-line chemotherapy start date should have occurred by the time of the latest date of metastatic diagnosis being made (30 days) and informed consent being given (90 days). The window to metastatic diagnosis date was defined to capture treatments given after the initial clinical/radiologic metastatic diagnosis

*BRCA* breast cancer susceptibility gene, *FAS* full analysis set, *gBRCAm* germline *BRCA* mutation

*If an agent was reported in two or more different regimens or treatment combinations in the first line, the agent was counted only once for that patient

The most frequently used cytotoxic chemotherapy agent as first-line therapy, either as a single agent or in combination, regardless of gBRCAm status, was paclitaxel (*n* = 127/341; 37.2%), followed by cyclophosphamide (*n* = 60/341; 17.6%), capecitabine (*n* = 57/341; 16.7%), docetaxel (*n* = 48/341; 14.1%), carboplatin (*n* = 31/341; 9.1%), doxorubicin (*n* = 29/341; 8.5%), and gemcitabine (*n* = 28/341; 8.2%) (Table 4). First-line cytotoxic chemotherapy regimens are detailed in Additional file 2.

## Discussion

The objectives of the BREAKOUT study were to estimate the true prevalence of gBRCAm in patients with HER2-negative metastatic breast cancer, by minimizing selection bias, and to generate observational outcome data. Outcome data, however, were not obtained from the BREAKOUT study, as the study was terminated early due to inadequate recruitment.

The estimate of gBRCAm prevalence in the observational BREAKOUT study was 9.7% (95% CI 6.8%, 13.3%) among a cohort of 341 patients with HER2-negative metastatic breast cancer being treated with first-line cytotoxic chemotherapy. Previous studies in unselected patient populations reported the prevalence of a g*BRCA1*m and a g*BRCA2*m to be between 1.2 and 8.8% [13–19]. In contrast, in a US study of 119 patients with HER2-negative metastatic breast cancer who were referred for mutation testing, and who likely had perceived risk factors for having a gBRCAm, the estimated prevalence was higher than that observed in the BREAKOUT population, at 24.4% [21]. Overall there were no notable differences in the prevalence of a gBRCAm with regards to HR status, sites of metastases, time from initial breast cancer diagnosis to entry into the BREAKOUT study, and physicians’ choice of first-line cytotoxic chemotherapy regimens.

In patients with at least one risk factor (family history of breast and/or ovarian cancer; age at initial breast cancer diagnosis ≤ 50 years; or triple-negative breast cancer), gBRCAm prevalence was 10.4% in the BREAKOUT study, compared with 5.8% in patients without any of these risk factors. Also of note is that approximately half of the patients with a gBRCAm in BREAKOUT (54.5%) did not have a family history of breast and/or ovarian cancer. This is notable as, although the number of patients involved is small, 5.8% is a sizeable proportion of patients whose gBRCAm could be missed if the criteria for gBRCAm testing in patients with HER2-negative metastatic breast cancer are based on the presence of risk factors, including family history of breast and/or ovarian cancer, as well as other tumor types such as prostate and pancreatic cancer [22]. Similar findings were observed in a US study in a population of patients with any-stage triple-negative breast cancer, in which 16% of patients with a gBRCAm did not have an established reason for gBRCAm testing and 10% had limited family history knowledge of breast and/or ovarian cancer at any age [23]. The data from the BREAKOUT study support the National Comprehensive Cancer Network recommendation that all patients with HER2-negative metastatic breast cancer undergo gBRCAm testing [24].

The prevalence of sBRCAm in 64 gBRCAwt patients with archival tumor tissue was 6.3% (one patient with s*BRCA1* and three with s*BRCA2*). The prevalence of any HRR gene mutation (excluding s*BRCA*) in this group was 7.8%. There are very limited data that describe the prevalence of somatic or germline non-*BRCA* HRR gene mutations in patients with metastatic breast cancer. Data from the Cancer Genome Atlas Program (TCGA) show that ∼ 20% of basal-like breast cancers have a germline and/or somatic *BRCA1* or *BRCA2* variant [25]. In the ongoing PRAEGNANT (Prospective Academic Translational Research Network for the Optimization of Oncological Health Care Quality in the Adjuvant and Advanced Therapeutic Setting) registry study in Germany (NCT02338167), of 1462 patients with metastatic breast cancer receiving any therapeutic regimen who had available germline DNA from time of study entry and successful genotyping, 4.3% had a non-*BRCA* germline HRR gene mutation [26]. Results from the PRAEGNANT study are expected to provide further insights into the prevalence of germline HRR gene mutations and their impact on outcome [27]. In addition, the European AURORA study that is recruiting 1300 patients with metastatic breast cancer will likely generate data on the prevalence of germline and somatic HRR gene mutations and their impact on treatment outcomes [28].

This study had some limitations that could impact the generalizability of the results. With the gBRCAm testing costs covered by this observational study, investigators may have been more likely to enroll patients who were at higher risk for having a gBRCAm. However, the patient characteristics of the BREAKOUT metastatic breast cancer population, including age, menopausal status, and HR status, were similar to those described in other HER2-negative metastatic breast cancer patient populations that were unselected for gBRCAm status [29–33]. An additional limitation was that the BREAKOUT study excluded patients previously treated with PARP inhibitors and, therefore, participating study sites were more likely to be those in which patients had not been exposed to PARP inhibitors in clinical trials. This may have had some influence on the patient population, as well as the size and type of participating centers, and this might have contributed to the inadequate recruitment.

The smaller than planned sample size, due to the early termination of the study, had an impact on the level of precision and generalizability of the findings. The study was designed to describe the prevalence of a gBRCAm with a precision of approximately ± 2% and, with only 341 patients in the FAS, a precision of 6.5% was achieved.

The BREAKOUT study incorporated the findings of gBRCAm tests performed prior to the study if they were available. To avoid distortion by a possible overrepresentation of patients with a prior test, participating sites were instructed to enroll patients in consecutive order, regardless of the availability of prior *BRCA* mutation test results. In the FAS, 8.8% (*n* = 30) of patients were tested for gBRCAm status prior to the baseline, and of these, 8 patients (26.7%) had a gBRCAm (these patients were repeat tested at baseline).

## Conclusions

The global BREAKOUT study demonstrated that 9.7% of patients with HER2-negative metastatic breast cancer who were receiving a first-line cytotoxic chemotherapy regimen had a gBRCAm and that 5.8% of the enrolled patients who had no standard risk factors had a gBRCAm.

The results help characterize the patient population who may benefit from PARP inhibitor therapy and highlight the need for broad gBRCAm testing of patients with HER2-negative metastatic breast cancer.

## Supplementary information

**Additional file 1: Table S1.** List of participating sites. This table details the sites that participated in the BREAKOUT study.

**Additional file 2: Table S2.** First-line cytotoxic chemotherapy regimens in > 5% of patients (FAS). This table details first-line cytotoxic therapy regimens (single agent and combination agent).

## Data Availability

Data underlying the findings described in this manuscript may be obtained in accordance with AstraZeneca’s data-sharing policy, described at https://astrazenecagrouptrials.pharmacm.com/ST/Submission/Disclosure

## Abbreviations

BC: Breast cancer
*BRCA*: Breast cancer susceptibility gene
CI: Confidence interval
CT: Chemotherapy
FAS: Full analysis set
gBRCAm: Germline *BRCA* mutation
gBRCAwt: Germline *BRCA* wildtype
HER2: Human epidermal growth factor receptor 2
HR: Hormone receptor
HRR: Homologous recombination repair
HRRm: Homologous recombination repair gene mutation
MBC: Metastatic breast cancer
OS: Overall survival
PARP: Poly(ADP-ribose) polymerase
PFS: Progression-free survival
PRAEGNANT: Prospective Academic Translational Research Network for the Optimization of Oncological Health Care Quality in the Adjuvant and Advanced Therapeutic Setting
sBRCAm: Somatic *BRCA* mutation
sHRRm: Somatic homologous recombination repair gene mutation
SD: Standard deviation
