# Supplementary material for: Prevalence of germline BRCA mutations in HER2-negative metastatic breast cancer: global results from the real-world, observational BREAKOUT study
Source: Breast Cancer Res. 2020 Oct 27;22:114. doi: 10.1186/s13058-020-01349-9 (PMC7590609; doi:10.1186/s13058-020-01349-9)
Supplement: Supplementary file 1 — Additional file 1: Table S1. List of participating sites. This table details the sites that participated in the BREAKOUT study. [file 13058_2020_1349_MOESM1_ESM.docx]

**Additional file 1**

**Table S1** List of participating sites

| Country | Center address | No. of consented patients |
| --- | --- | --- |
| Australia | Ballarat Base Hospital, Drummond Street North, Ballarat, 3350, Victoria, Australia | 1 |
| Australia | Redcliffe Hospital, Anzac Avenue, Redcliffe, 4020, Queensland, Australia | 2 |
| Australia | Ashford Cancer Centre Research, 520 South Road, Suite 10, Tennyson Centre, Kurralta Park, 5037, South Australia, Australia | 3 |
| Bulgaria | Complex Oncological Center-Ruse EOOD, 2, Nezavisimost Str., Ruse, 7002, Bulgaria | 5 |
| Bulgaria | MHAT - Dobrich, AD, 24 Panayot Hitov Str., Dobrich, 9300, Bulgaria | 2 |
| Bulgaria | Complex Oncological Center - Ruse, EODD, 2, Nezavisimost Str., Ruse, 7002, Bulgaria | 8 |
| Bulgaria | Acibadem City Clinic Tokuda Hospital EAD, 51B, Nikola Vapcarov Blvd., Sofia, 1407, Bulgaria | 3 |
| Canada | Cancer Centre of Southeastern Ontario at Kingston General Hospital, 25 King Street West Cancer Centre of Southeastern Ontario, Kingston, K7L 5P9, Ontario, Canada | 3 |
| Canada | Grand River Hospital, 835 King Street W PO Box 9056, Kitchener, N2G 1G3, Ontario, Canada | 2 |
| Canada | CHU de Quebec -Hôpital du Saint-Sacrement, 1050 Chemin Ste-Foy, Québec, G1S 4L8, Quebec, Canada | 2 |
| Canada | Centre Intégré Universitaire du Saguenay–Lac-Saint-Jean, 305 Rue St Vallier, Chicoutimi, G7H 5H6, Quebec, Canada | 2 |
| Hungary | Jasz-Nagykun-Szolnok Megyei Hetenyi Geza Korhaz-Rendelointezet, Toszegi ut 21, Szolnok, 5004, Hungary | 1 |
| Hungary | Szegedi Tudomanyegyetem Szent-Gyorgyi Albert Klinikai Kozpont, Koranyi Fasor 12, Szeged, 6720, Hungary | 1 |
| Hungary | Tolna Megyei Balassa Janos Korhaz, Beri Balogh Adam u. 5-7, Szekszard, 7100, Hungary | 1 |
| Hungary | Magyar Honvedseg Egeszsegugyi Kozpont, Podmaniczky u. 109-111, Budapest, 1062, Hungary | 4 |
| Italy | Fondazione Maugeri Pavia, Via Salvatore Maugeri, 8-10, Pavia, 27100, Pavia, Italy | 4 |
| Italy | Arcispedale S. Maria Nuova Azienda Ospedaliera di Reggio Emilia, Viale Risorgimento 80, Reggio Emilia, 42100, Reggio Emilia, Italy | 1 |
| Italy | Ospedale Ramazzini di Carpi, Via Molinari, 2, Carpi, 41012, Modena, Italy | 1 |
| Italy | Casa di Cura Multimedica Ospedale di Castellanza, Viale Piemonte, 70, Castellanza, 21053, Varese, Italy | 1 |
| Italy | Azienda Socio Sanitaria Territoriale Niguarda (Grande Ospedale Metropolitano Niguarda), Piazza Ospedale Maggiore, 3, Milano, 20162, Milano, Italy | 1 |
| Japan | Kitano Hospital, The Tazuke Kofukai Medical Research Institute, Kita-ku Ogi-machi 2-4-20, Osaka-shi, 530-8480, Osaka-Fu, Japan | 3 |
| Japan | NHO Hokkaido Cancer Center, Shiroishi-ku Kikusui 4jo 2-3-54, Sapporo-shi, 003-0804, Hokkaido, Japan | 8 |
| Japan | Tohoku University Hospital, Aoba-ku Seiryo-machi 1-1, Sendai-shi, 980-8574, Miyagi-Ken, Japan | 6 |
| Japan | St. Luke's International Hospital, Akashi-cho 9-1, Chuo-ku, 104-8560, Tokyo-To, Japan | 4 |
| Japan | NHO Shikoku Cancer Center, Minamiumemoto-machi Ko 160, Matsuyama-shi, 791-0280, Ehime-Ken, Japan | 12 |
| Japan | NHO Kyushu Cancer Center, Minami-ku Notame 3-1-1, Fukuoka-shi, 811-1395, Fukuoka-Ken, Japan | 4 |
| Japan | Tesshokai Kameda General Hospital, Higashi-cho 929, Kamogawa-shi, 296-8602, Chiba-Ken, Japan | 8 |
| Korea, Republic of | Ulsan University Hospital, 877, Bangeojinsunhwan-doro Dong-gu, Ulsan, 44033, Korea, Republic of | 10 |
| Korea, Republic of | Asan Medical Center, 88, Olympic-ro 43-gil, Songpa-gu Professor office, West building 8F, Seoul, 05505, Korea, Republic of | 7 |
| Korea, Republic of | National Cancer Center, 323, Ilsan-ro, Ilsandong-gu, Goyang-si, 10408, Gyeonggi-do, Korea, Republic of | 5 |
| Korea, Republic of | Seoul National University Bundang Hospital, 82 Gumi-ro 173beon-gil Bundang-gu, Seongnam-si, 13620, Gyeonggi-do, Korea, Republic of | 6 |
| Korea, Republic of | Severance Hospital, Yonsei University Health System, 134 Shinchon-Dong, Seodaemun-Gu Clinical Trial Pharmacy, 2F, Jejung building, Seoul, 120-752, Korea, Republic of | 5 |
| Korea, Republic of | Seoul National University Hospital, 101 Daehak-ro, Jongno-gu, Seoul, 03080, Korea, Republic of | 3 |
| Korea, Republic of | Gangnam Severance Hospital, Yonsei University Health System, 211, Eonjuro, Gangnam-gu, Seoul, 06273, Korea, Republic of | 5 |
| Korea, Republic of | CHA Bundang Medical Center, CHA University, 59, Yatap-ro, Bundang-gu, Seongnam-si, 13496, Gyeonggi-do, Korea, Republic of | 3 |
| Korea, Republic of | Dong-A University Hospital, 26, Daesingongwon-ro, Seo-gu IRB, Dong A University Hospital, Busan, 49201, Korea, Republic of | 4 |
| Korea, Republic of | Chung-Ang University Hospital, 102 Heukseok-ro Dongjak-gu, Seoul, 06973, Korea, Repblic of | 2 |
| Poland | Szpital Specjalistyczny W Brzozowie, Podkarpacki Osrodek Onkologiczny Im.Ks.B.Markiewicza, ul. ks. Bielawskiego 18, Brzozow, 36-200, Poland | 2 |
| Poland | Opolskie Centrum Onkologii im.prof.T.Koszarowskiego, ul. Katowicka 66 A, Opole, 45-060, Poland | 2 |
| Poland | Dolnoslaskie Centrum Onkologii, Pl. Hirszfelda 12, Wroclaw, 53-413, Poland | 4 |
| Poland | Magodent Szpital Elblaska, ul. Szamocka 6, Warszawa, 01-748, Poland | 3 |
| Poland | Specjalistyczny Szpital im. dr A. Sokolowskiego, ul. Sokolowskiego 4, Walbrzych, 58-309, Poland | 2 |
| Poland | Mazowiecki Szpital Onkologiczny, ul. Koscielna 61, Wieliszew, 05-135, Poland | 1 |
| Poland | Onko-Dent G.L. Słomian SP.J., ul. Centralna 17, Zory, 44-240, Poland | 1 |
| Russian Federation | Tomsk Research Institute of Oncology, 12/1, Savinykh Str., Tomsk, 634028, Russian Federation | 1 |
| Russian Federation | BHI of Omsk region “Clinical Oncology Dispensary”, 9/1, Zavertyaeva str., Omsk, 644013, Russian Federation | 17 |
| Russian Federation | SBIH of Stavropol territory "Pyatigorsk Oncological Dispensary", 31, Kalinina str., Pyatigorsk, 357502, Russian Federation | 1 |
| Russian Federation | SBHI of Novosibirsk Region "Novosibirsk Regional Oncological Dispensary", 2, Plakhotnogo str., Novosibirsk, 630108, Russian Federation | 2 |
| Russian Federation | DCH on Station Chelyabinsk of JSC "Russian Railways", 41, Tsvillinga str., Chelyabinsk, 454091, Russian Federation | 5 |
| Russian Federation | SBIH " Clinical Oncological Dispensary # 1", 146, Dimitrova str., Krasnodar, 350040, Russian Federation | 1 |
| Russian Federation | LLC Evimed, 9-V, Blukhera str. 10, Chelyabinsk, 454048, Russian Federation | 3 |
| Russian Federation | SBI of Ryazan region "Regional Clinical Oncological Dispensary", 13, Sportivnaya str., Ryazan, 390011, Russian Federation | 3 |
| Russian Federation | LEC at CJSC "Avicenna", Kommunisticheskaya Ul., 17/1, Novosibirsk, 630099, Russian Federation | 2 |
| Spain | Complejo Hospitalario Universitario A Coruña, Hospital Teresa Herrera (Materno-Infantil) C/Xubias de Abaixo s/n, A Coruña, 15006, La Coruña, Spain | 5 |
| Spain | Hospital de Terrassa, Ctra. Torrebonica s/n, Terrassa, 08227, Barcelona, Spain | 1 |
| Spain | ICO Girona - Hospital Universitari de Girona Dr. Josep Trueta, Avda. de França, s/n Oncología, Girona, 17007, Girona, Spain | 1 |
| Spain | Hospital Universitario Fundacion Alcorcon, c/ Budapest, 1 Cardiologia, Alcorcon, 28922, Madrid, Spain | 2 |
| Spain | Hospital Universitari Vall d'Hebron, Passeig Vall d'Hebron, 119-129, Vall d'Hebron University Hospital Servicio de Oncologia, Edifici Modul Blau - Planta baixa, Edificio Modulares Azules, Barcelona, 08035, Barcelona, Spain | 1 |
| Spain | Hospital Universitario de Burgos, Avda. Islas Baleares, 3, Burgos, 09006, Burgos, Spain | 2 |
| Spain | Hospital Universitario Infanta Sofia, Paseo de Europa, 34 Servicio de Oncologia, Planta 2 (Hospital de día), San Sebastian de los Reyes, 28702, Madrid, Spain | 2 |
| Spain | Complejo Hospitalario de Especialidades Juan Ramon Jimenez, Ronda Norte s/n, Huelva, 21005, Huelva, Spain | 5 |
| Taiwan, Republic of China | Changhua Christian Hospital, 135 Nan-Hsiao Street, Changhua, 50004, Taiwan, Republic of China | 1 |
| Taiwan, Republic of China | Chi Mei Medical Center, Liou Ying, No. 201, Taikang, Taikang Vil., Liuying Dist., Tainan, 736, Taiwan, Republic of China | 5 |
| Taiwan, Republic of China | China Medical University Hospital, 2 YuDe Road, Taichung, 40447, Taiwan, Republic of China | 1 |
| Taiwan, Republic of China | Kaohsiung Veterans General Hospital, No. 386, Dazhong 1st Rd., Zuoying Dist., Kaohsiung, 81362, Taiwan, Republic of China | 3 |
| Taiwan, Republic of China | Tri-Service General Hospital, 325 Sec 2 Cheng-Kung Road, Neihu District, Taipei, 11490, Taiwan, Republic of China | 4 |
| Taiwan, Republic of China | National Cheng Kung University Hospital, 138, Sheng-Li Rd., Tainan, 704, Taiwan, Republic of China | 2 |
| Taiwan, Republic of China | E-Da Hospital, No.21, Yida Road Yanchao Township, Kaohsiung, 824, Taiwan, Republic of China | 1 |
| Taiwan, Republic of China | Cheng-Hsin Rehabilitation Medical Center, No.45, Cheng Hsin St., Pai-Tou, Taipei, 112, Taiwan, Republic of China | 4 |
| Turkey | Medical Park Samsun Hastanesi, Mimar Sinan Mah. Alparslan Bulvarı, No. 17 Atakum, Medical Park Samsun Hastanesi, Samsun, 55200, Turkey | 7 |
| Turkey | Dicle University, Medical Faculty, Dicle Universitesi Tip Fakultesi Ic Hastaliklari Anabilim Dali Medikal Onkoloji Bilim Dali, Sur / Kampus, Diyarbakir, 21080, Turkey | 15 |
| Turkey | Istanbul Medeniyet Univ Goztepe Training & Res Hosp, Istanbul Medeniyet Univ. Goztepe Egitim ve Arastirma Hastanesi Tıbbi Onkoloji Bilimdali, D100 Karayolu Merdivenkoy Mevkii Goztepe Kardiyoji Bolumu, Goztepe, Istanbul, 34854, Turkey | 2 |
| Turkey | Acibadem Adana Hospital, Acibadem Adana Has. Cumhuriyet Caddesi, Adana, 01130, Turkey | 11 |
| Turkey | Izmir Medicalpark Hospital, İzmir Medical Park Hastanesi Yeni Girne Bulvarı 1825 Sok. No:12, Karşıyaka / İzmir, Izmir, 35530, Turkey | 7 |
| Turkey | Akdeniz University Medical Faculty, Akdeniz Universitesi Tip Fakultesi Ic Hastaliklari Anabilim Dali Tibbi Onkoloji Bilim Dali Dumlupinar Bulvari, Antalya, 07058, Turkey | 5 |
| Turkey | Dr. Abdurrahman Yurtaslan Oncology Teaching and Research Hospital, Dr. Abdurrahman Yurtaslan Onkoloji Egitim ve Arast. Hastanesi Seflik Binasi 2, Onkoloji Klinigi 12, Cadde Demetevler, Ankara, 06105, Turkey | 5 |
| Turkey | Sakarya Training and Research Hospital, Sakarya Egitim ve Arastirma Hastanesi Tibbi Onkoloji Bilimdali, Esentepe Kampusu, Sakarya, 54187, Turkey | 7 |
| Turkey | Kocaeli Universitesi Tip Fakultesi, Kocaeli Universitesi Tip Fak. Umuttepe Merkez Yerleskesi, Onkoloji Anabilimdali, Kocaeli, 41380, Turkey | 2 |
| Turkey | Dr. Lutfi Kirdar Kartal Training and Research Hospital, Egitim ve Arastirma Hastanesi Ic Hastaliklari Klinigi Semsi Denizer Cad. E-5 Karayolu Cevizli Mevkii, Kartal, Istanbul, 34890, Turkey | 2 |
| Turkey | Karadeniz Tecnical Uni. Med. Fac., Karadeniz Teknik Universitesi Tip Fak Farabi Hastanesi Trabzon Merkez, Trabzon Merkez, Trabzon, 61100, Turkey | 3 |
| Turkey | Yuzuncu Yil University Medical Faculty, Yuzuncu Yil Universitesi Tip Fakultesi Medikal Onkoloji Bilim Dali Zeve Kampusu, Van, 65080, Turkey | 1 |
| Turkey | Baskent University Adana Application and Research Center, Baskent Universitesi Adana Dr. Turgut Noyan Uygulama ve Arastirma Hastanesi Dadaloglu Mah. 2591 Sok. No. 4A, Adana, 01250, Turkey | 5 |
| Turkey | Yildirim Beyazit University Ankara Ataturk Training and Research Hospital, Universiteler Mah., Bilkent Caddesi No. 1 Yildirim Beyazit Universitesi Ankara Ataturk E.A.H., Cankaya, Ankara, 6800, Turkey | 4 |
| Turkey | Ankara Numune Train.&Res. Hosp, Ankara Numune EAH Hacettepe Mahallesi Talatpasa Bulvari No. 44, Altindag, Ankara, 06100, Turkey | 5 |
| Turkey | Namik Kemal University, Namik Kemal Universitesi Tip Fakultesi Psikiyatri Anabilimdali Yuzuncu Yil Mah. Ugur Mumcu Cad., Itfaiye Binasi Arkasi, Tekirdag, 59100, Turkey | 1 |
| Turkey | Istanbul Onkoloji Hastanesi - Tıbbi Onkoloji, Cevizli Mah. Toros Cad. No. 86 Maltepe, Istanbul, 34846, Turkey | 2 |
| UK | Royal Stoke University Hospital, The Cancer Centre Newcastle Road, Stoke on Trent, ST4 6QG, Staffordshire, UK | 2 |
| UK | University College London Hospitals, 250 Euston Road 1st Floor east, London, NW1 2PG, Greater London, UK | 1 |
| UK | Royal Derby Hospital, Uttoxeter Road, Derby, DE22 3NE, Derbyshire, UK | 1 |
| UK | Peterborough City Hospital, Edith Cavell Campus Bretton Gate, Peterborough, PE3 9GZ, Cambridgeshire, UK | 6 |
| UK | Hinchingbrooke Hospital, Hinchingbrooke Park, Huntingdon, PE29 6NT, Cambridgeshire, UK | 5 |
| UK | Worthing Hospital, Lyndhurst Road, Worthing, BN11 2DH, East Sussex, UK | 3 |
| UK | Royal Cornwall Hospital, Treliske, Truro, TR1 3LJ, Cornwall, UK | 3 |
| UK | Huddersfield Royal Infirmary, Acre Street Lindley, Huddersfield, HD3 3EA, West Yorkshire, UK | 2 |
| UK | New Cross, Wolverhampton Road, Wolverhampton, WV10 0QP, West Midlands, UK | 1 |
| UK | Blackpool Victoria Hospital, Whinney Heys Road, Blackpool, FY8 1OW, Lancashire, UK | 2 |
| UK | Warwick Hospital, Lakin Road, Warwick, CV34 5BW, Warwickshire, UK | 1 |
| UK | Royal Devon and Exeter Hospital (Wonford), Barrack Road Wonford, Exeter, EX2 5DW, Devon, UK | 3 |
| UK | Royal Lancaster Infirmary, Ashton Road, Lancaster, LA1 4RP, Lancashire, UK | 4 |
| USA | Wenatchee Valley Hospital & Clinics, 820 N Chelan Ave, Wenatchee, 98801, Washington, USA | 3 |
| USA | Peninsula Cancer Institute, 12100 Warwick Blvd Suite 201, Newport News, 23601, Virginia, USA | 1 |
| USA | Texas Oncology, P.A. - Paris, 3550 Northeast Loop 286, Paris, 75460, Texas, USA | 1 |
| USA | Texas Oncology, P.A. - Denton, 3720 south I-35 East, Denton, 76210, Texas, USA | 1 |
| USA | Texas Oncology, P.A. - Flower Mound, 4370 Medical Arts Drive Suite 100, Flower Mound, 75028, Texas, USA | 1 |
| USA | Baylor Charles A. Sammons Cancer Center, 3410 Worth Street Suite 540, Dallas, 75246, Texas, USA | 9 |
| USA | Compass Oncology, 210 SE 136th Avenue Compass Oncology Vancouver, Vancouver, 98684, Washington, USA | 2 |
| USA | Santa Barbara Hematology Oncology Medical Group, Inc., 540 West Pueblo Street, Santa Barbara, 93105, California, USA | 2 |
| USA | Texas Oncology - Memorial City, 925 Gessner Road, Houston, 77024, Texas, USA | 3 |
| USA | Rocky Mountain Cancer Centers, LLP, 1800 Williams Street, Suite 100, Denver, 80218, Colorado, USA | 3 |
| USA | Texas Oncology-San Antonio Northeast, 2130 NE Loop 410 Suite 100, San Antonio, 78217, Texas, USA | 3 |
| USA | Gulf Coast Cancer and Diagnostic Cancer Center, 11281 Beamer Road, Houston, 77089, Texas, USA | 1 |
| USA | Specialist Global Research, 1490 West 49th Place Suite 505, Hialeah, 33012, Florida, USA | 3 |
