# Supplementary material for: Prevalence of germline BRCA mutations in HER2-negative metastatic breast cancer: global results from the real-world, observational BREAKOUT study
Source: Breast Cancer Res. 2020 Oct 27;22:114. doi: 10.1186/s13058-020-01349-9 (PMC7590609; doi:10.1186/s13058-020-01349-9)
Supplement: Supplementary file 2 — Additional file 2: Table S2. First-line cytotoxic chemotherapy regimens in > 5% of patients (FAS). This table details first-line cytotoxic therapy regimens (single agent and combination agent). [file 13058_2020_1349_MOESM2_ESM.docx]

**Additional file 2**

**Table S2**  First-line cytotoxic chemotherapy regimens in >5% of patients (FAS)

|  | **gBRCAm status** | |  |
| --- | --- | --- | --- |
|  | **Positive  (N=33)** | **Negative (N=308)** | **FAS (N=341)** |
| **Single agent regimens, (%)** |  |  |  |
| n | 15 | 181 | 196 |
| Paclitaxel | 4 (26.7) | 71 (39.2) | 75 (38.3) |
| Capecitabine | 5 (33.3) | 37 (20.4) | 42 (21.4) |
| Docetaxel | 2 (13.3) | 22 (12.2) | 24 (12.2) |
| Cyclophosphamide | 1 (6.7) | 11 (6.1) | 12 (6.1) |
| **Combination agent regimens, n (%)^*^** |  |  |  |
| n | 18 | 127 | 145 |
| Paclitaxel/bevacizumab | 4 (22.2) | 17 (13.4) | 21 (14.5) |
| Paclitaxel/carboplatin | 3 (16.7) | 11 (8.7) | 14 (9.7) |
| Carboplatin/gemcitabine | 1 (5.6) | 11 (8.7) | 12 (8.3) |
| Doxorubicin/cyclophosphamide | 0 | 12 (9.4) | 12 (8.3) |
| Cisplatin/gemcitabine | 0 | 9 (7.1) | 9 (6.2) |
| Cyclophosphamide/epirubicin + hydrochloride | 2 (11.1) | 6 (4.7) | 8 (5.5) |

FAS, full analysis set

^*^11 patients reported multiple agents or combinations of agents without a progression event; only the first combination of agents used are listed
First-line cytotoxic chemotherapy was defined as the first chemotherapy given in the metastatic setting up to disease progression. First-line cytotoxic chemotherapy start date occurred on the latest date of (metastatic diagnosis date – 30 days) and (informed consent date – 90 days). The 30 days window to metastatic diagnosis date was defined to capture treatments given after the initial clinical/radiologic metastatic diagnosis
